# Recovering signals in physiological systems with large datasets

**DOI:** 10.1242/bio.019133

**Published:** 2016-07-21

**Authors:** Hodjat Pendar, John J. Socha, Julianne Chung

**Affiliations:** 1 Department of Biomedical Engineering and Mechanics, Virginia Tech Blacksburg, Blacksburg, VA 24061, USA; 2 Department of Mathematics, Virginia Tech Blacksburg, Blacksburg, VA 24061, USA

**Keywords:** Input estimation, Deconvolution, Ill-conditioned inverse problems, Flow-through respirometry

## Abstract

In many physiological studies, variables of interest are not directly accessible, requiring that they be estimated indirectly from noisy measured signals. Here, we introduce two empirical methods to estimate the true physiological signals from indirectly measured, noisy data. The first method is an extension of Tikhonov regularization to large-scale problems, using a sequential update approach. In the second method, we improve the conditioning of the problem by assuming that the input is uniform over a known time interval, and then use a least-squares method to estimate the input. These methods were validated computationally and experimentally by applying them to flow-through respirometry data. Specifically, we infused CO_2_ in a flow-through respirometry chamber in a known pattern, and used the methods to recover the known input from the recorded data. The results from these experiments indicate that these methods are capable of sub-second accuracy. We also applied the methods on respiratory data from a grasshopper to investigate the exact timing of abdominal pumping, spiracular opening, and CO_2_ emission. The methods can be used more generally for input estimation of any linear system.

## Abstract (precis)

**Summary:** We introduce two methods to recover true physiological signals from indirectly measured data. As a demonstration, we show precise recovery of gas-exchange patterns from a grasshopper recorded in a flow-through respirometry system.

## INTRODUCTION

Real-time measurements of endogenous and exogenous signals in living organisms are widely important in fields that span biology and medicine. However, the signals of interest are often not directly accessible, and their effect can only be measured indirectly. Hormone secretion, rate of glucose utilization, oxygen consumption, and CO_2_ production are just a few examples of such signals that cannot be directly measured (Bartholomew et al., 1981; Veldhuis et al., 1987; De Nicolao et al., 1997). A common approach to deal with this problem is to sample intermittently and then perform data fitting. For example, to determine hormone secretion or glucose production rate, blood concentration is repeatedly measured, and then rates are estimated from the recorded sequence (Veldhuis et al., 1987). However, the uncertainty of these estimates can be high due to the ill-conditioning of the systems. An ill-conditioned problem is an inherently unstable problem, where small errors in the data can lead to large errors in the solution, thereby making accurate estimation of the true signal very challenging (Engl et al., 1996; Hansen, 2010).

**Fig. 1.**
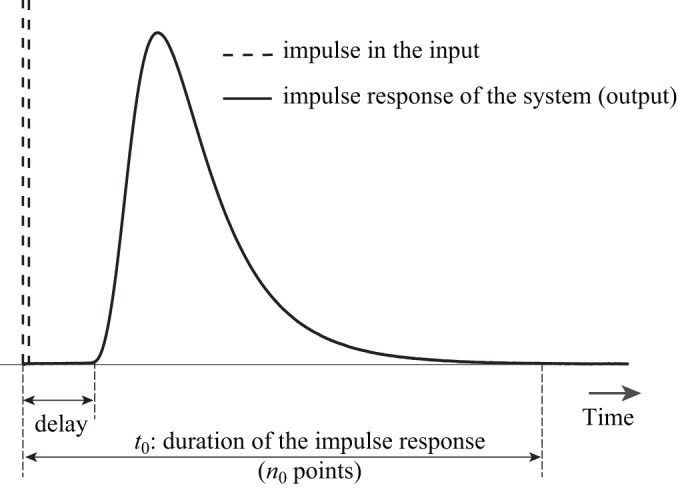
**Impulse response of the system.** When a short pulse of CO_2_ is injected into the chamber and the concentration of the gas outside of the chamber is recorded with a gas analyzer, the shape of the output signal will be different from the input signal. This output signal is called the impulse response of the respirometry system.

Measuring gas exchange in animals is an important example of indirect measurement of physiological signals. To determine the gas exchange rate of an animal using flow-through respirometry, the animal is put in a respirometry chamber and air is flowed through the chamber. The flowed air continuously replaces the consumed oxygen by the animal, washes out the CO_2_ and water vapor produced by the animal, and brings the gases to a gas analyzer. The pattern of measured gas concentration in the gas analyzer can be substantially different than the pattern of real instantaneous gas exchange of the animal, depending on the washout dynamics of the system (Lighton and Halsey, 2011; Lighton, 2012). Any short burst of CO_2_ gradually leaves the respirometry system over the duration of a few seconds to several hours (Fig. 1). During this time interval, the short burst can also be combined with other CO_2_ bursts. Therefore, interpretations of physiological function based on raw respiratory data can be misleading (Gray and Bradley, 2006), and can become even more problematic if the respiratory signal has to be synced with another real-time and rapidly changing signal such as pressure, movement, or temperature.

Generally, these physiological measurement problems, in addition to many other unrelated scientific applications such as image de-blurring and 3D tomographic reconstruction, are referred to as ‘input-estimation’ or ‘inverse problems' (De Nicolao et al., 1995, 1997; Chung and Nagy, 2010; Chung et al., 2011). In an input estimation problem, the signal of interest, represented by the input signal *u*, is not available, but a transformation or convolution of *u*, called the output signal *y*, is measurable. Assuming that the system is linear and time-invariant, the output signal (*y*) can be written as a convolution of the input signal (*u*) and the impulse response of the system (Hespanha, 2009):
(1)
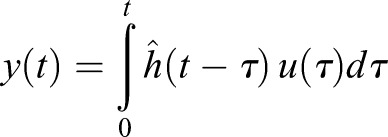
where *y*(*t*) is the output of the system at time *t*, which is measured by the gas analyzer. 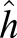 is the impulse response of the system, which can be found experimentally (Fig. 1) (Pendar and Socha, 2015). *u* is the true gas exchange signal from the animal, which we aim to recover. The discrete form of this convolution equation is given by:
(2)
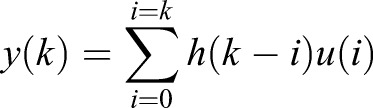
where *y*(*k*) and *u*(*k*) describe the average of the output and input respectively, when *kδt*≤*t*<(*k+1*)*δt*, where *δt* is the sampling interval and *h*(*k*) is the integral of in the sampling period:
(3)
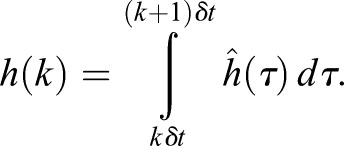
Eqn 2 can be represented using the matrix equation:
(4)
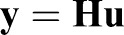
where 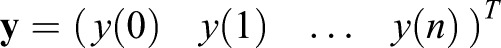 is the measured output of the system, 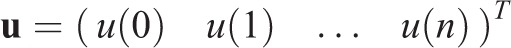 is the desired, unknown input, and
(5)
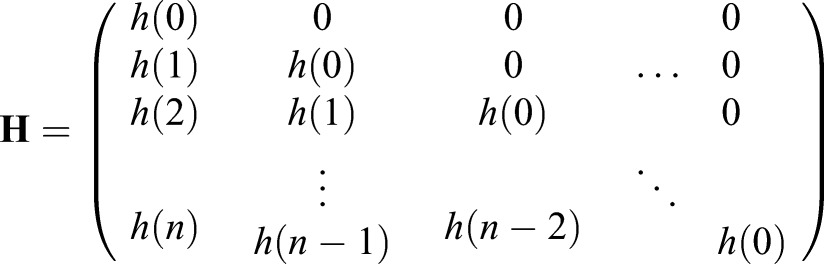
models the forward convolution process. Although deconvolution can be modeled as a linear equation (Eqn 4), solving this system is not trivial due to the ill-posedness of the underlying problem (Neumaier, 1998). This is revealed in the inherent singularity and poor conditioning of matrix **H**. Furthermore, the solution is very sensitive to noise in the measured data and computational errors (De Nicolao et al., 1997). That is, small errors in the data can lead to large errors in the solution, and the inverse solution **H**^−1^**y** is useless. Regularization methods have been proposed to remedy ill-conditioning by solving a related problem that is both uniquely solvable and robust to noise (Engl et al., 1996; Hansen, 2010). Tikhonov regularization is a well-known and widely used approach to solve such inverse problems (Golub et al., 1999). However, efficient implementations for very large datasets, such as when measurements are taken frequently and/or over long time intervals, are challenging. In this paper, we propose two approaches for extending Tikhonov regularization to large datasets. First, we take advantage of the small duration of the impulse response relative to the size of the dataset to partition the dataset and apply the Tikhonov method in a sequential manner. Second, we introduce a dimension reduction approach to regularization that reduces the ill-conditioning of the system and uses an averaging of least-squares solutions to recover the inputs of the original system.

### Background

#### Tikhonov method

Tikhonov regularization has been used to solve a variety of inverse problems such as image de-blurring, tomographic reconstruction, and estimation of gas exchange (Tikhonov et al., 1977; De Nicolao et al., 1997; Vicini et al., 1997; Tokuyama et al., 2009; Chung and Nagy, 2010). Although other methods such as trend-identification, Kalman filtering, and Kalman smoothing can be used for input estimation, the Tikhonov method is often preferred for its better performance in gas exchange problems, for example in studies with whole-room calorimeters (Tokuyama et al., 2009).

The Tikhonov solution is given by
(6)
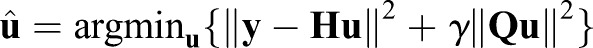


where γ>0 is a regularization parameter and **Q** is a design or regularization matrix that enforces smoothness of the solution. The first term on the right side of Eqn 6 is the data fit term, which measures how loyal the estimate is to the data, and the second term penalizes roughness of the solution. **Qu** usually represents the discrete derivative of **u**. Therefore, the solution of Eqn 6 tends to be smooth. The closed-form solution of the above equation is:
(7)
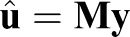
where
(8)
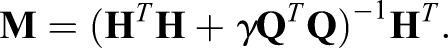
The regularization parameter γ plays an important role in the quality of the Tikhonov solution and is typically tuned via an experimental calibration. A large value of γ puts more emphasis on the regularization term and makes the solution smoother, thereby preventing the solution from being corrupted by inverted noise. On the other hand, a small value of γ puts less weight on the regularization term, and the solution resembles the noise-contaminated inverse solution. Various methods have been proposed to choose γ, such as the discrepancy principle, the L-curve, the unbiased predictive risk estimator, and the generalized cross-validation method (Hunt, 1972; De Nicolao et al., 1997; Hansen, 2010). Some of these methods require prior knowledge about the noise.

There is also a variety of choices for the regularization matrix **Q** (De Nicolao et al., 1997; Tokuyama et al., 2009). In this area, the most common choices for **Q** include the identity matrix (**Q**=**I**) or a discretization of the derivative operator. For example, for respirometry, **Q** can be a lower triangle Toeplitz matrix, where the first column is given by (1−2 1 0 … 0)^*T*^, which penalizes the sum of squared second differences. If penalizing the second difference causes excessive smoothness (e.g. the estimation loses sharp changes in the input), **Q** can be chosen to be a lower triangle Toeplitz matrix, where the first column is (1−1 0 …0)^*T*^, corresponding to the first difference of the estimation.

## RESULTS

Here, we introduce two new methods for input estimation.

### Method 1 - Extension of the Tikhonov method for large datasets

For very large datasets, computing the Tikhonov solution as in Eqn 7 and Eqn 8 may be computationally infeasible. In particular, because the dimensions of matrix **H** correspond to the size of the data vector, constructing and storing **H** and computations with **H** may be burdensome and expensive. Iterative methods may provide an alternative approach for large, sparse linear systems, but preconditioning may be required for fast convergence. Instead, we propose to apply the Tikhonov method to smaller fragments or partitions of the data, while being attentive to the overlapping regions. From the impulse response function, we know that any input signal, such as a short burst of CO_2_, will contribute to future output/measurements for the duration of *t*_0_, meaning for *n*_0_ data points (Fig. 1). Therefore, the input between 0 to *t* (*n* data points) affects the output from 0 to *t*+*t*_0_ (*n*+*n*_0_ data points). To recover the input, we propose the following algorithm:

1: Consider the first *n*_0_+*n* data points and construct corresponding matrices **H**, **Q** and, 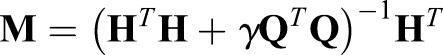. Notice that these are small matrices of size *n*+*n*_0_, so the inverse or matrix factorizations (e.g., QR or LU) can be easily computed.
2: Use Eqn 7 to estimate the *n*+*n*_0_ inputs from the first *n*+*n*_0_ data points (***y***). Because the last *n*_0_ input points affect the output points that are not included in the first *n*+*n*_0_ output points, only accept and record the first *n* inputs or less, and disregard the rest of the estimated inputs.
3: To deal with overlapping regions, use Eqn 2 or Eqn 4 to determine the corresponding output for the first *n* estimated inputs, and then subtract it from the data vector ***y***. Eliminate the first *n* points of the new **y** vector and repeat the sequence from (2) again.

This algorithm does not determine all the inputs at once, but rather estimates *n* points of the input at each cycle. The computational advantage of this algorithm is that 1) it does not require the inverse of a large matrix, and 2) for equally spaced partitions, matrix **M** or an efficient representation of **M** only needs to be computed once. The main computational cost per cycle is a matrix-vector multiplication with the inverse, or a simple linear solve, which is very cheap. The regularization parameter γ must be determined experimentally for each custom setup.

### Method 2 - Dimension reduction method

Another approach to deal with ill-conditioning or the singularity of **H** is to use a projection approach or dimension reduction technique. The basic idea is the assumption that the solution lies in a low-dimensional subspace, which has a regularizing effect on the problem (i.e., eliminating the singularity and/or significantly improving the conditioning of the problem). Here, assume that *m* consecutive points in the input signal are equal:
(9)
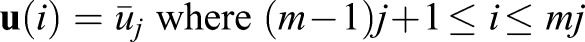


If we assume, without loss of generality, that the number of data points 
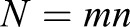
, then the projected problem has the form,
(10)
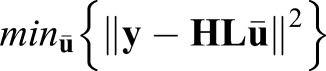


where 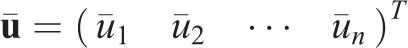 is an *n*×1 vector and **L** is a *N*×*n* matrix with the following elements:
(11)
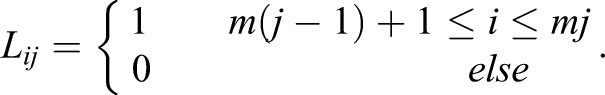


Because of the added constraint on the input vector, the projected problem is better conditioned than the original problem and Eqn 10 can be solved using any least-squares method for over-determined systems of equations, because **HL** has full column rank. In summary, the solution in the original space is given by
(12)
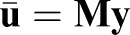
 where
(13)
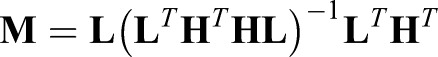


Because we have assumed that *m* consecutive input points are equal, the solution in Eqn 12 cannot track input changes within these intervals, and false artifacts such as sharp jumps between intervals are introduced as a consequence of this assumption. To improve the accuracy of the solution and to obtain a smoother representation, we slide the interval *m* times in increments of 1, each time computing an estimate of the input, and we take the final solution to be the average of these *m* solutions. More precisely, let 1≤*k*≤*m* and let **P**_*k*_ be a lower shift matrix whose (*i*, *j*)^*th*^ component is: 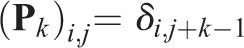, where *δ* is the Kronecker delta. For example, **P**_*k*_**X** shifts the components of vector **X** down by 1 element and introduces a zero in the first element, whereas 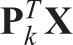 shifts the components of vector **X** up by 1 element and introduces a zero in the last element. Then, for each *k*, a solution can be computed as

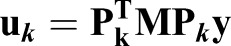
where **M** is defined in Eqn 13. By linearity, the average of all *m* solutions is given by
(14)
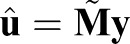
where:
(15)
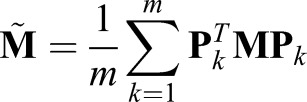


Thus, each reconstruction matrix is just a shifted submatrix of the original matrix, and important spectral properties can be shown.

For extremely large data sets, we can combine the dimension reduction approach with the partitioning approach described in method 1. That is, we can apply this method on *n*_0_+*n* data points, accept only the first *n* estimated inputs, find the corresponding output of the *n* estimated input points, and subtract it from the vector **y**. Then, we eliminate the first *n* points of the data and repeat the procedure for the next *n*_0_+*n* data points.

### Numerical experiments and validation

**Fig. 2.**
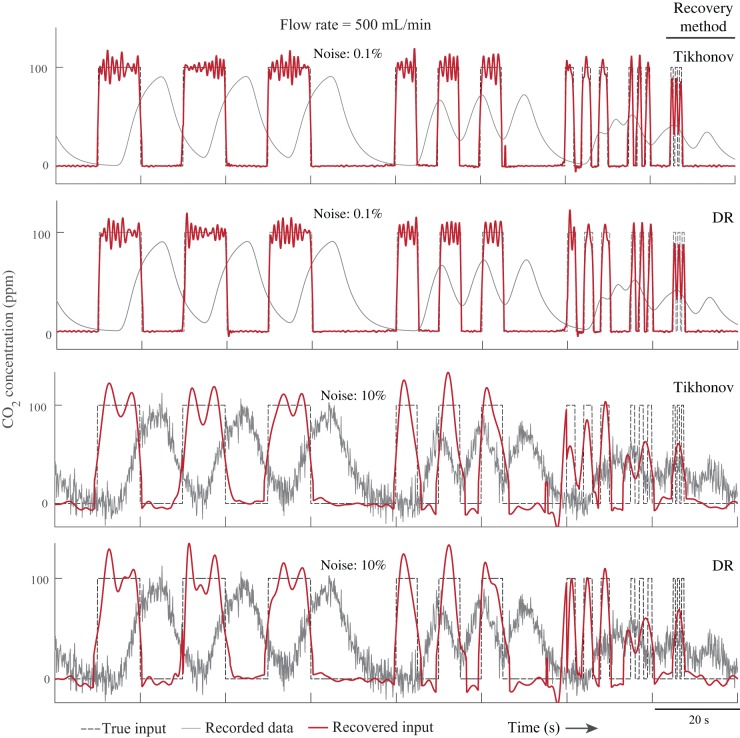
**Simulation results (flow rate: 500 ml/min).** Both Tikhonov and dimension reduction (DR) methods were able to recover fast changes in the input (1 Hz or 0.5 s pulses) when the noise level is low. With extremely noisy data (10%), the methods were able to recover pulses with duration of 2 s.

**Fig. 3.**
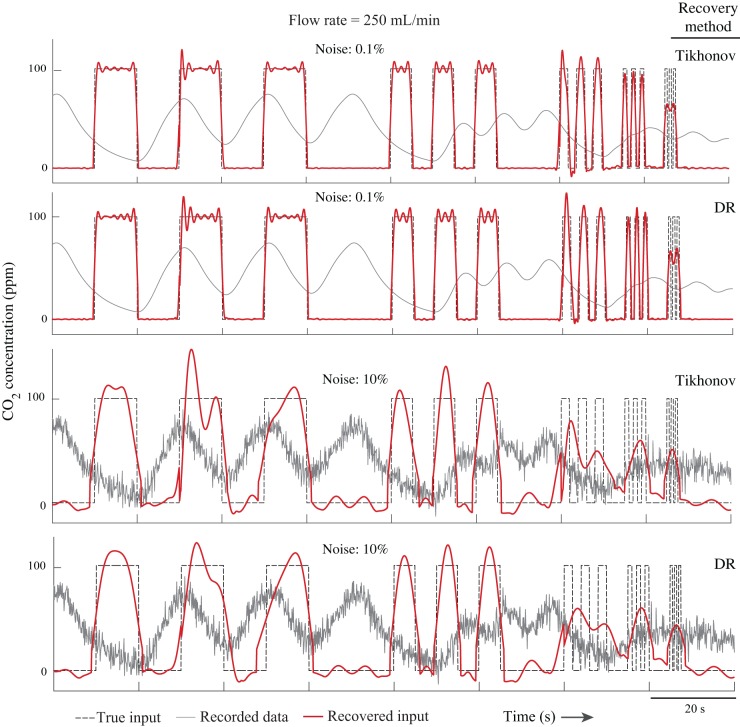
**Simulation results (flow rate: 250 ml/min).** When the noise level was low, the methods recovered the inputs with the frequency of 0.5 Hz (pulse duration of 1 s). With extremely noisy data (10%) the methods recovered 0.1 Hz (pulse duration of 5 s) input signals.

To computationally validate the methods, we simulated a respirometry system and found the output gas concentration for an arbitrary instantaneous gas exchange pattern (see Materials and Methods for details). The input signals were recovered using both the extension of the Tikhonov method and dimension reduction method and compared with the true input signal. The results of both methods are very similar (Figs 2, 3 and Tables 1, 2). By increasing the input frequency and noise, the accuracy of the methods decreased. The methods captured pulses with durations down to 0.5 s in the higher flow rate (*F*=500 ml/min), but only when the noise was less than 0.1% (Fig. 2). Both methods recovered the pulses with durations down to 2 s when the noise level was up to 5%, which is a very high value. In the higher flow rate, the 2 s pulses were recovered even with a high noise level of 10% (Figs 2, 3; see Fig. S1 for more detail). In general, by decreasing the tuning parameters (*γ* in Tikhonov method and *m* in dimension reduction method), it is possible to recover even higher frequency inputs; however, the results become noisier. The Pearson correlation coefficient of the recovered signals and the true signals varied by noise level (Tables 1, 2), and these numbers further indicate that both methods perform very closely.

**Table 1.**
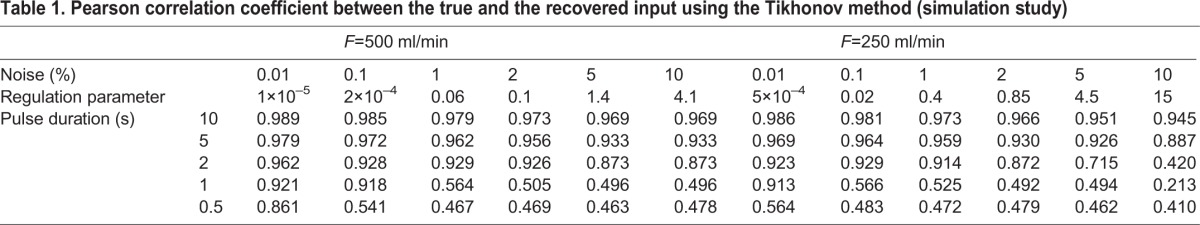
**Pearson correlation coefficient between the true and the recovered input using the Tikhonov method (simulation study)**

**Table 2.**
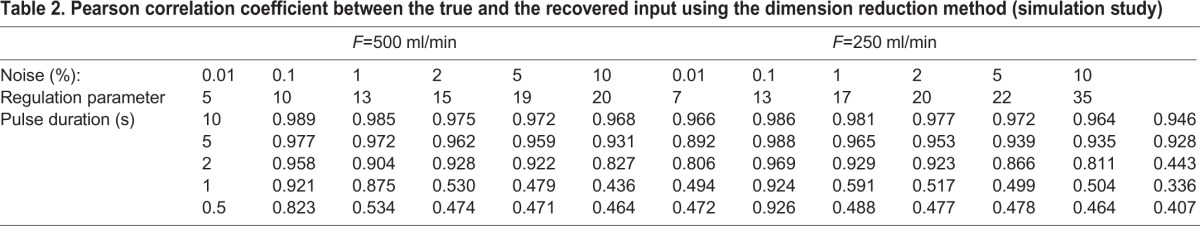
**Pearson correlation coefficient between the true and the recovered input using the dimension reduction method (simulation study)**

### Experimental validation

**Fig. 4.**
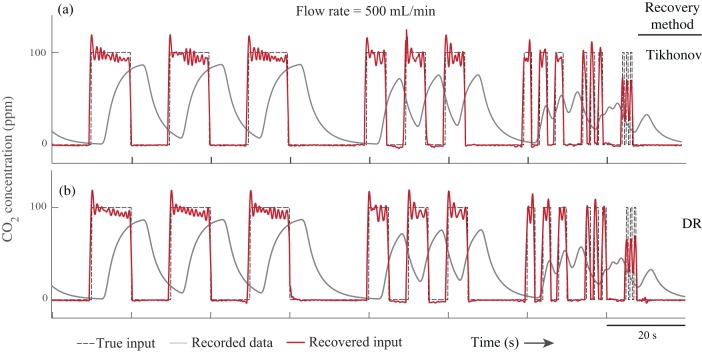
**Experimental results (flow rate: 500 ml/min).** Both Tikhonov and dimension reduction (DR) methods were able to recover fast changes in the input with the frequency of 1 Hz or pulse duration of 0.5 s.

**Fig. 5.**
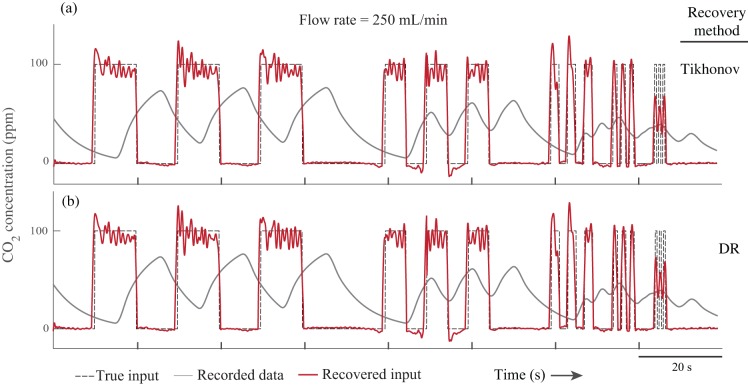
**Experimental results (flow rate: 250 ml/min).** Even in this lower flow rate, both Tikhonov and dimension reduction methods successfully recovered the fast changes in the input (frequency of 1 Hz).

We also tested the methods experimentally by perfusing CO_2_ with an arbitrary pattern into a respirometry chamber and recorded the output concentration of CO_2_ with a gas analyzer. Then we applied the methods to recover the true CO_2_ injection pattern (see Materials and Methods for the details). The noise level of the respirometry system was measured to be 0.014%, determined by recording the CO_2_ concentration using the same methodology, but with an empty chamber. The estimations of instantaneous gas exchange rate from both methods are very similar (Figs 4, 5). The Pearson correlation coefficients between the recovered signal and the true signal for different input frequencies, different flow rates, and signal recovery methods were calculated separately (Tables 1, 2). The difference between the Pearson correlation coefficients for the two methods is 0.02% on average. Both methods were able to recover pulses with duration of 0.5 s in both flow rates; however, both were more accurate with the higher flow rate of 500 ml/min (Figs 4, 5 and Table 3).

**Table 3.**
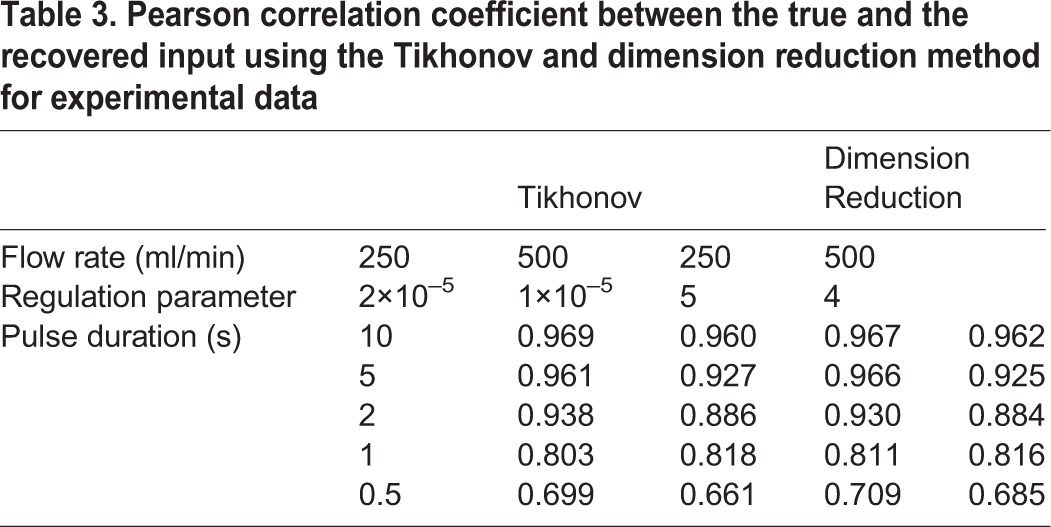
**Pearson correlation coefficient between the true and the recovered input using the Tikhonov and dimension reduction method for experimental data**

### Case study: abdominal pumping, spiracular control, and CO_2_ emission in grasshopper

To demonstration of the effectiveness of the methods on a real biological system, we measured the CO_2_ production rate of a male grasshopper (*Schistocerca americana*) in a flow-through respirometry system. We also recorded the abdominal movements and the movements of the second thoracic spiracle on the left side of the body with a videocamera. The developed methods were applied to the CO_2_ data to recover the instantaneous respiratory pattern, and this signal was then synced with the abdominal and spiracular movement signals (see Materials and Methods for the details). The duration of the abdominal pumps and CO_2_ bursts of the grasshopper were 1.9±0.4 s and 1.1±0.3 s, respectively. Most abdominal pumps (67.4%) were followed by the opening of the thoracic spiracle. All openings of the thoracic spiracle started when the abdomen began to relax at the end of the abdominal pump. In contrast, the spiracle was closed during the start of the pump cycle. The duration of the spiracle's open phase was 0.5±0.1 s (mean±s.d.).

**Fig. 6.**
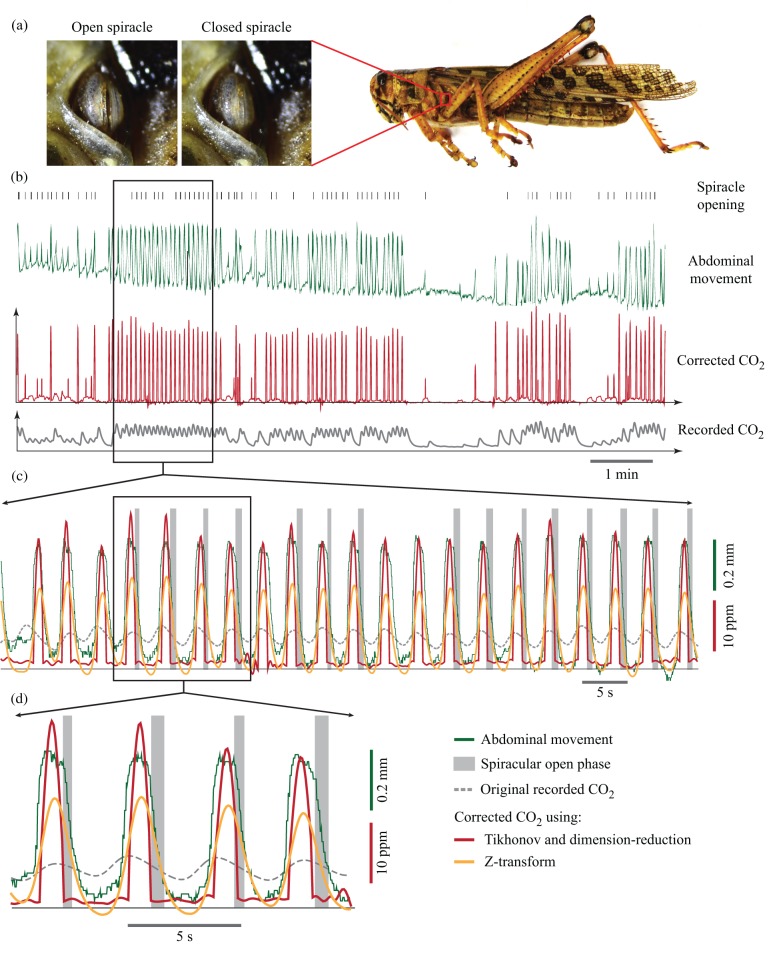
**Unidirectional airflow in grasshoppers.** (A) In this experiment, abdominal pumping, spiracular state, and CO_2_ emission in a grasshopper were recorded and synchronized together. (B,C) The raw CO_2_ signal shows a cyclic pattern of respiration. However, the corrected CO_2_ signals shows discontinuous gas exchange, indicated by periods of CO_2_ release. (D) The recovered CO_2_ signal (corrected using the Tikhonov method) shows that abdominal pumping was concurrent with the CO_2_ emission, but almost all the CO_2_ burst happened when the thoracic spiracle was closed. The spiracles open when the abdomen starts to relax. The recovered CO_2_ signal corrected using the Z-transform method did not reveal these details.

The raw data of the CO_2_ emission show a continuous pattern of gas exchange. However, the corrected CO_2_ emission signal reveals that the animal was exhibiting discontinuous gas exchange (Fig. 6) with periods of thoracic spiracular closure, followed by bursts of CO_2_ release. All CO_2_ bursts coincided with abdominal pumps on a one-to-one basis. The correlation coefficient of the abdominal movement and the raw CO_2_ signal was 0.376; however, after recovering the true CO_2_ signal, this value increased to 0.73. There were also some abdominal pumps and CO_2_ bursts observed that were not followed by the opening of the spiracle (28.4%). Based on the raw CO_2_ signal, only 44.7% of the CO_2_ was emitted during abdominal pumping; however, the corrected CO_2_ signal shows that 76.5% of the CO_2_ was released during abdominal pumping. There was only a small amount of CO_2_ emitted during the opening phase of the thoracic spiracle (8.6%), and most of the CO_2_ was released when the spiracle was closed.

## DISCUSSION

Here, we extended the well-known Tikhonov method for large-scale problems using a partitioning approach, and also presented a new dimension reduction method that reduces a large ill-conditioned problem to a set of smaller, better-conditioned problems. Both methods were tested computationally and experimentally on a flow-through respirometry system. Based on our computational and experimental evaluations, these methods exhibit nearly identical performance.

Although the accuracies of both methods in recovering the true signal were similar, the dimension reduction method is computationally more efficient. In both methods, an inverse of a matrix must be calculated (Eqns 8 and 13). In the Tikhonov method, the inverse of **H**^*T*^**H**+*γ***Q**^*T*^**Q** must be calculated, and the size of **H** is bigger than the size of the impulse response. Therefore, it could be a large matrix. However, in the dimension reduction method, the inverse of **L**^*T*^**H**^*T*^**HL** must be determined (Eqn 13), and its size is *m* times smaller. The factor *m* will be different integers for different experimental setups. In our work, after experimentally tuning *m*, this number fell between 4 and 30 for different experiments and simulations with a variety of noise levels. Therefore, in these experiments and simulations, the size of the matrix was 4 to 30 times smaller in comparison to that of the matrix for the Tikhonov method on the original problem. Considering the large size of this matrix and that both methods use the inverse of this matrix, having a 4 to 30 times smaller size can decrease the computational cost, particularly when the size of the impulse response (and consequently the size of the **H** matrix) is very large.

Previously, it has been shown that the Tikhonov method exhibits much better performance in comparison with other methods such as trend identification, Kalman filtering, and Kalman smoothing (Tokuyama et al., 2009). However, applying the Tikhonov method requires calculating the inverse of a large matrix, with a size equal to the length of the data, or many iterations of a numerical optimization scheme. In contrast, the extension of the Tikhonov method and the dimension reduction methods that we provide in this paper are applicable to large datasets of any size. The methods are reasonably straightforward and have only one parameter to be tuned. This feature makes them less complicated to use than the generalized Z-transform (GZT) method, recently introduced by our group (Pendar and Socha, 2015), which requires the use of multiple parameters that must be determined experimentally. Therefore, tuning of the Tikhonov method is simpler.

**Fig. 7.**
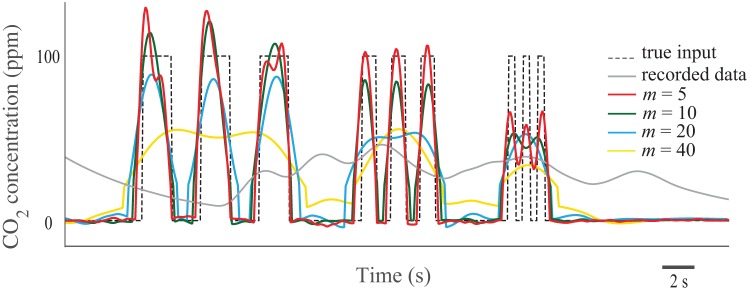
**The effect of the tuning factor (*m*) in the performance of recovery.** By choosing a small number for *m* in the dimension reduction method, the fast dynamical changes in the input signal can be recovered. However, the results could become noisy.

Both of the new methods introduced here rely on a single parameter that should be tuned for best results: γ in the Tikhonov method, and *m* in the dimension reduction method. By increasing the tuning parameter, each method becomes less sensitive to noise in the data, but this comes at the cost of losing fidelity to the true input signal. In particular, these methods lose accuracy in estimating sharp changes of the input. Conversely, by decreasing the tuning parameter, the methods become better in capturing fast changes in the input, but this comes at the cost of becoming more sensitive to noise and errors (Fig. 7). For standard Tikhonov regularization, many parameter-selection methods have been developed and investigated for automatically choosing the parameter; this includes the discrepancy principle and generalized cross-validation (Vainikko, 1982; Engl et al., 1996; Colton et al., 1997). In practice, however, it is often desirable to tune the parameter via trial and error, based on the goals of the experiment. For instance, if determining fast changes in the input is crucial and some small amount of noise in the solution can be tolerated, a researcher can use a smaller γ in the Tikhonov method or *m* in the dimension reduction method. For the dimension reduction approach, we have not developed a method that can be used to determine the optimal value of *m*, and this remains a target for future study. In our simulations and experiments, we chose it by trial and error.

One of the possible problems in the dimension reduction method is that the matrix **L**^*T*^**H**^*T*^**HL** in Eqn 13 becomes singular or nearly singular, making the inversion unstable. By the nature of their design, flow-through respirometry systems have a delay between the signal input and the measured output (τ in Fig. 1) (Lighton, 2008). This phenomenon appears as *n*_0_ zeroes at the beginning of the impulse response (*h*) and results in *n*_0_ rows of zeroes in the matrix **H**. This can cause a singularity in **H**^*T*^**H** and **L**^*T*^**H**^*T*^**HL** in Eqns 9 and 15. In the Tikhonov method (Eqn 8), the regularization term *γ***Q**^*T*^**Q** is able to deal with the singularity. For the dimension reduction method (Eqn 14), we propose two methods to prevent singularity, even if the *n*_0_ elements of the impulse response are forced to be zero:

1. We eliminate the delay and therefore the singularity by eliminating the *n*_0_ zeros from the beginning of the impulse response, and eliminate the first *n*_0_ data points from the output. After eliminating these points, the **H** matrix should be constructed based on the new impulse response, and the new output vector (*y*) should be used in Eqn 15. Applying this modification will not change the solution.
2. Similar to the Tikhonov method, we can add a regularization term (*γ***Q**^*T*^**Q**) to the projected problem. In this way, Eqn 14 changes to: **M**=**L**(**L**^*T*^**H**^*T*^**HL**+*γ***Q**^*T*^**Q**)^−1^**L**^*T*^**H**^*T*^.

### The importance of the methods in physiological studies

In some studies, precisely determining the true timing of the gas exchange signal is crucial. In general, it becomes more important if we want to synchronize the gas exchange data with another fast dynamical event, such as a change in body temperature (Bartholomew and Barnhart, 1984), body movement (Parkes et al., 2002; Pendar et al., 2015), tracheal deformation (Socha et al., 2008), or even heartbeat (Woakes and Butler, 1983). To demonstrate the effectiveness of the methods on a real physiological system, we synchronized the CO_2_ signal of a grasshopper with its abdominal and spiracular movements, both of which include relatively fast dynamics, with durations of the second or sub-second timescale. Therefore, the true CO_2_ signal must be recovered with sub-second accuracy if we are to understand the relationship amongst the signals.

Grasshoppers possess 10 pairs of spiracles with two pairs on the thorax and eight pairs on the abdomen. The timing and pattern of spiracular opening and closing can vary wildly, depending on the metabolic needs of the animal (Miller, 1960b; Harrison, 1997; Harrison et al., 2013). Resting but alert grasshoppers normally exhibit abdominal pumping, driven by dorsoventral expiratory muscles (Hustert, 1975). If some of the spiracles are open, the contraction of the abdomen causes some parts of the tracheal system to compress (Harrison et al., 2013), forcing the air out of the system. During exhalation, the first four posterior pairs of spiracles close, and expiration occurs through the last six pairs (Lewis et al., 1973; Hustert, 1975). When the abdomen relaxes, spiracles 5-10 close and spiracles 1-4 open, and an increase in the volume of the tracheal system causes bulk flow of air into the body through the anterior spiracles (Fraenkel, 1932; Miller, 1960b). This spiracular timing during abdominal pumping helps to produce a unidirectional flow of air from anterior to posterior within the tracheal system (Miller, 1960b).

A unidirectional flow of air is not unique to grasshoppers, and has been reported in beetles, bees, and cockroaches as well (Bailey, 1954; Duncan and Byrne, 2002; Heinrich et al., 2013). However, the determination of unidirectionality has been based on the observation of average air movement from the thorax to the abdomen side over a long period (Fraenkel, 1932; Bailey, 1954; Duncan and Byrne, 2002). In these studies, an animal was put in a chamber with a barrier to separate the external air from the abdominal and thoracic sides. Over the course of minutes to hours, it is observed that the air gradually moves from the thorax to the abdomen side. Miller (1960a,b) also measured the abdominal movement and status of four spiracles simultaneously to infer the direction of flow. In his experiments, CO_2_ was not measured, and expiration and inspiration were interpreted from the abdominal movement. However, we now know that abdominal pumping can be decoupled with respiration (Groenewald et al., 2012; Pendar et al., 2015), so this inference can be wrong. In our experiment, we simultaneously measured abdominal movement, spiracle movement, and CO_2_ emission. The CO_2_ signal recovery methods enabled the true respiratory signal to be discerned with sub-second resolution, which in turn enabled their synchronization with two other real-time signals. This helped us to directly observe, for the first time, the relationship between the true individual CO_2_ expiration bursts, abdominal pumping, and the status of a spiracle in a grasshopper.

Knowing the timing of the spiracles and how they coordinate with abdominal pumping, tracheal deformation, gas exchange, and other respiratory relevant signals is of fundamental importance to understanding the underlying mechanistic basis of active ventilation in insect respiration. However, measuring these events simultaneously can be difficult. Our data (Fig. 6) show that when the grasshopper pumps its abdomen, it closes the thoracic spiracle and releases CO_2_, which indicates that some of the other spiracles must be open within this duration. When the insect relaxes the abdomen, it opens the thoracic spiracle, but there is almost no CO_2_ emitted in this period, which suggests that the grasshopper is drawing the air in during this period. These observations are congruent with previous observations about active ventilation (Greenlee et al., 2009) and the unidirectionality of the airflow in grasshoppers (McCutcheon, 1940; Weis-Fogh, 1967).

The Z-transform method, also known as the Bartholomew method, has perhaps been most widely used to correct gas exchange signals, but the temporal resolution of this method is not always sufficient to study fast changes in respiratory signals (Pendar and Socha, 2015). We compared the results of the grasshopper experiment using our new methods with those using the Z-transform method (Fig. 6). As interpreted, the corrected CO_2_ signal using the Z-transform method (Fig. 6) would indicate the presence of CO_2_ emission during all spiracular open phases, a quantitatively different interpretation than what was obtained with our new methods. Using the Z-transform method, we would not have been able to clearly identify the evidence for unidirectional flow, demonstrating the power of the new methods.

In any respirometry system, the washout problem reduces the accuracy of the recorded respiratory data, obscuring details of gas exchange. The issue becomes more problematic when we want to find the relationship between gas exchange and other fast dynamical events. Our demonstration with the grasshopper was only one example to show the importance of finding the true respiratory signal with fast dynamics, but the methods are broadly applicable for other types of respiratory studies. For example, they could be usefully applied in studies of the discontinuous gas exchange cycle (DGC), a topic of heavy debate (Schneiderman, 1960; Schneiderman and Schechter, 1966; Lighton, 1996; Shelton and Appel, 2001; Chown et al., 2006). DGC involves three phases: (1) the closed phase, in which the insect keeps the spiracles closed and consumes oxygen from the air sealed inside the tracheal system, with CO_2_ buffered in the hemolymph; (2) the flutter phase, in which the spiracles open slightly and then close rapidly in sequence; and (3) the open phase, in which the insect keeps the spiracles open and releases CO_2_. In many studies, the open phase is reported to last for several minutes to hours (Hetz and Bradley, 2005; Chown et al., 2006). But, to the best of our knowledge, there is no explanation for the long duration of the open phase. The hygric (Buck et al., 1953), chthonic (Lighton and Berrigan, 1995), and oxidative damage (Hetz and Bradley, 2005) hypotheses are the main proposed explanations for DGC, explaining that insects keep their spiracles closed for a long time to decrease water loss, to maximize the partial pressure gradient between the tracheal system and environment, or to keep the oxygen level down inside the tracheal system to minimize the tissue damage, respectively. None of these hypotheses can explain why insects keep their spiracles open for a long time during the open phase. Keeping the spiracles open for a long duration can be detrimental for multiple reasons: it could lead to considerable water loss, decrease the partial pressure gradient, and/or expose the tissues to high levels of oxygen for a long time. However, our results from the grasshopper trial suggest that it might be possible that, during the so-called open phase, the spiracles are not actually open continuously. Instead, the spiracles may in fact close intermittently, but because of the washout issue and insufficient input estimation, the CO_2_ signal wrongly indicates a continuous CO_2_ emission, a point that has been previously suggested (Gray and Bradley, 2006). This behavior is congruent with discontinuous gas exchange of cockroaches, which have been observed to exhibit multiple opening and closing of spiracles in the open phase (Kestler, 1984). Based on our observations in this study, we also suggest that any interpretation from the raw CO_2_ signal, or even the Z-transformed signal, could be misleading without using an accurate recovery method such as the presented methods in this paper.

### Conclusion

We have developed two new methods for accurately estimating the input signal in any physiological system. Our test case with a grasshopper demonstrates that these methods can be used to extract high temporal information for the original signal, which can dramatically change the interpretation of the underlying physiological processes. Although the Tikhonov method is widely used for signal processing applications across fields, few researchers have employed it for input estimation of physiological systems. The novelty of our modification of the Tikhonov method is that this extension makes it possible to apply the method to datasets of any size, including very large datasets. Furthermore, our new dimension reduction method can produce quality solutions that are similar to Tikhonov solutions, but in a slightly more efficient manner. This method has the potential to be beneficial in other applications such as image processing.

To assist a researcher in implementing these methods for their own studies and to encourage further development of the methods, we provided detailed instructions and the code as an open source resources at https://github.com/TheSochaLab/Extended-Tikhonov-and-Dimension-Reduction-methods-for-data-recovery. For researchers who may be less familiar with such methods, we also provided detailed instruction.

Overall, these improvements in input estimation have the potential to change the way physiologists view indirectly recorded data, most particularly for studies of gas exchange.

## MATERIALS AND METHODS

### Evaluation of the methods

The new methods presented here were evaluated both in simulation and experiment.

#### Numerical experiments and validation

A MATLAB code (available at https://github.com/TheSochaLab/Extended-Tikhonov-and-Dimension-Reduction-methods-for-data-recovery) was written to model a flow-through respirometry system using Eqn 1. This code determines the output (**y**) of the respirometry system for any given input (**u**) and impulse response (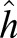). We used the impulse responses of a rectangular 28 ml flow-through respirometry chamber model with inlet flow rates of 250 and 500 ml/min, which were determined experimentally in a previous study (Pendar and Socha, 2015). The input was considered to be sequences of three rectangular pulses with different frequencies of 0.1, 0.2, 0.5, 1 and 2 Hz (Figs 2, 3). The simulation was repeated by adding 0.01%, 0.1%, 1%, 2%, 5%, and 10% normally-distributed noise to the output. The sampling rate of the signals was assumed to be 10 Hz, with the duration of the virtual experiment of one hour (36,000 data points). Both methods were used to recover the input from the output signal. Because the size of the dataset was large, we applied both methods to the data points, *n*+*n*_0_=1500, at each iteration (*n*_0_=720, Fig. 1). The recovered inputs were compared with the true input at each frequency and each noise level separately.

#### Experimental validation

To test the methods experimentally, we used a high-speed valve (MHE2-MS1H-5/2-M7-K, Festo, NY, USA) to switch between CO_2_ gas (100 ppm, balanced with N_2_) and regular air immediately before a 28 ml (25×25×45 mm^3^) respirometry chamber similar to the simulation setup. The experiment was repeated with two inlet flow rates of 250 ml/min and 500 ml/min. Data were recorded for one hour with a sampling rate of 10 Hz. Both reconstruction methods were applied to the recorded data to recover the original CO_2_ infusing pattern. The details of the experimental setup are described in Pendar and Socha (2015).

#### Case study: abdominal pumping, spiracular control, and CO_2_ emission in a grasshopper

In insects, air enters the body through valves called spiracles, and then is directly delivered to the tissues through a complex network of tracheal tubes. The same system is used to transport CO_2_ from the tissues to the surrounding air. Gas transport occurs via mixed diffusion and advective flows (Socha et al., 2008). These flows can be created by compression of the tracheal system, which can be linked to abdominal pumping and an increase in hemolymph (blood) pressure (Pendar et al., 2015). The pattern of airflow can also be affected by the spiracular valve timing. Thus, studying the coordination of abdominal pumping, hemolymph pressure, the status of spiracles, and CO_2_ emission is essential to understanding how insects actively breathe, whose record is embedded in the pattern of CO_2_ emission. However, the recorded data depends on many parameters of the experimental respirometry system, including flow rate, chamber volume, length of tubing, and internal structure of the gas analyzer, and emitted CO_2_ signals are not captured independently and in real time (Lighton and Halsey, 2011; Pendar and Socha, 2015). In contrast, hemolymph pressure, the status of the spiracles, and the movement of the abdomen can be measured or observed directly and in real time. To synchronize these signals, it is necessary to recover the instantaneous gas exchange from the CO_2_ record. Because the dynamics of the hemolymph pressure, abdominal movement, and spiracles can be fast, the instantaneous gas exchange must be determined with high temporal accuracy to be synched with other signals. Otherwise, timing patterns may be misinterpreted.

As a demonstration of the effectiveness of our methods on a real system, we measured the CO_2_ production rate of a male grasshopper (*Schistocerca americana*; mass=1340 mg) in a flow-through respirometry system at room temperature (22°C). For these measurements, the grasshopper was placed in a 28 ml chamber and CO_2_–free air was flowed in at rate of 500 ml/min; emitted CO_2_ was recorded with an infrared gas analyzer (LI 7000 Li-Cor, Nebraska, USA). The abdominal movements and the movements of the second thoracic spiracle on the left side of the body were also recorded with a video camera (NEX-VG10, Sony) at 30 frames per second (Movie 1). A total of 12 min were recorded, representing 100 abdominal pumping cycles. To determine the impulse response of the system, a simple model of the animal was made with adhesive putty and was placed inside the chamber. A short pulse of 100 ppm CO_2_ (duration, 200 ms) was injected into the chamber. The output of the gas analyzer was recorded and normalized to determine the impulse response of the system (Pendar and Socha, 2015). Lastly, we used the impulse response to define the forward model **H** and used it in both methods to recover the instantaneous CO_2_ emission of the animal.

The mesothoracic spiracles of grasshoppers are externally visible, so it was possible to determine the open/close status of this spiracle from the video records (Fig. 6). The lip-like spiracle contains two moving parts, the atrial lips, which together act as a valve. Using Quicktime software, video of the thoracic spiracle (Movie 1) was analyzed frame by frame to determine the open/closed status of the spiracle. Any frames that indicated a gap between the lips were coded as ‘open’. Correspondingly, when there was no gap between the lips, the frames were coded as ‘closed’. To determine the abdominal movement, we used a custom MATLAB code to track one point on the ventral cuticle frame by frame in the same movie. To synchronize the video data with the CO_2_ data, we first recorded a pulse of light initiated by a voltage pulse from the data acquisition system, and then aligned the signals post hoc.

### Regularizing properties of the dimension reduction method

Here, our goal is to provide justification and insight for the dimension reduction method. We first interpret the method as a projection approach, which is a form of regularization (Hansen, 2010).

Let **H** be an *N*×*N* convolution matrix of the form in Eqn 5, and **L** be an *N*×*n* matrix with entries given in Eqn 11. By construction, columns of **L** are orthogonal, and a simple scaling by 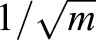 makes them orthonormal. Because the columns of **L** form an orthonormal basis, the projected problem seeks an approximation to the solution that lies in a low-dimensional subspace of R^N^. That is, Eqn 9 constrains the solution to a low-dimensional subspace, which has a regularizing effect on the solution. Other common projection approaches are based on Krylov subspace methods (Hansen, 2010).

The improved conditioning of the projected system, compared to the original system, can be explained by comparing matrices **H** and **HL**. First, notice that columns of **HL** represent averages of neighboring columns in the Toeplitz matrix **H**. Thus, if the columns of **H** are nearly linearly dependent, the columns of **HL** will be linearly independent. This is revealed in the rank of a matrix, which corresponds to the number of linearly independent columns. By properties of rank of a matrix, we have

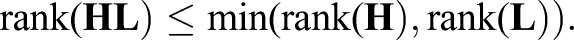
Typically, the rank of **H** is large (close to *N*) and **L** has full rank by construction, so we can assume that for sufficiently large *m*, **HL** is full rank (with rank *n*). A least-squares problem with an overdetermined full rank system has a unique solution that satisfies the normal equations (Ascher and Greif, 2011), which is used in our approach.

In the context of inverse problems, the above interpretation of the dimension reduction method as a constraint on the solution makes sense in terms of regularization. It is worth noting that matrix **L** and its transpose strongly resemble prolongation and restriction operators used in the multigrid literature (Briggs et al., 2000), but our approach is not a multigrid approach.

### Deriving equation 15 and spectral relationships

Here, we will derive Eqn 15, and discuss spectral relationships among the reconstruction matrices. The dimension reduction method initially assumes that *m* consecutive inputs are equal, leading to the projected problem in Eqn 9 and the computed solution in Eqn 12. However, with this assumption, the solution cannot recover input changes during the intervals, so we improve the method by incrementally sliding the intervals *m* times and taking the final solution to be an average of the *m* solutions. Let 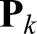 be a lower shift matrix whose (*i*,*j*)*^th^* component is: 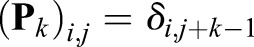, where *δ* is the Kronecker delta. For example, 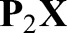 shifts the components of vector **X** down by 1 element and introduces a zero in the first element, whereas 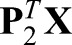 shifts the components of vector **X** up by 1 element and introduces a zero in the last element.

Thus to determine the *k*^th^ solution (1≤*k*≤*m*), we first shift the output vector down by *k*−1 elements, which can be represented by matrix multiplication, 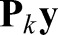. Here, we assume all outputs before time zero are zero (*y*_*j*_=0 if *j*<*k*−1). Then, we use the dimension reduction approach to compute a solution, 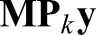. Finally, we shift the solution back up by *k*−1 elements, giving the kth solution, 
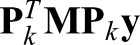
.

Although this solution is not precise for last *k* entries of the input vector, we eliminate the last *n*_0_ estimated inputs, so the lack of precision is inconsequential. Due to linearity of matrix multiplication, the average of all estimations is given as:
14
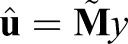
where:

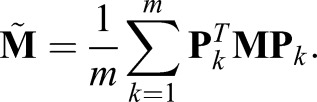
Notice that because 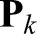
are shift matrices, 
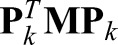
 can be obtained by shifting rows of **M** up *k*−1 times and columns of **M** left *k*−1 times, and filling the remaining entries with zero. This is computationally easy to perform.

### Using the codes

In addition to sample data and impulse response files, two open-source MATLAB codes are provided at https://github.com/TheSochaLab/Extended-Tikhonov-and-Dimension-Reduction-methods-for-data-recovery so that a user can perform the extended Tikhonov and the dimension reduction methods with their own experimental data. To calibrate the methods, it is necessary to find the impulse response of the system; this has been explained in detail previously in Pendar and Socha (2015). Briefly, to find the impulse response in a flow-through respirometry system, a short pulse of CO_2_ should be injected into the respirometry chamber, and this injection should be recorded concurrently with the data recorded by the gas analyzer. The recording should be continued until the CO_2_ signal completely vanishes. The recorded signal is the impulse response of the system, and it is specific to each custom respirometry setup (i.e., tubing connection, respirometry chamber, flow rate, etc.). The signal should be saved as a text file with the name ‘ImpulseResponse.txt’. This file should contain two columns: time and CO_2_ concentration. The sampling rate should be as same as the data-recording rate during the experiments. After finding the impulse response, the experimental data from the organism of interest can be corrected using the two methods described here. The experimental results should be saved in a text file with the name ‘Data.txt’ and it should contain two columns: time and gas concentration. The executed code recovers the true signal and saves it as a text file with the name ‘Recovered_Tikhonov.txt’ or ‘Recovered_DimentionReduction.txt’, based on the method of choice. The output file contains three columns: time, original signal, and the corrected signal.
